# Supplementary material for: Dynamic DNA methylation turnover in gene bodies is associated with enhanced gene expression plasticity in plants
Source: Genome Biol. 2023 Oct 12;24:227. doi: 10.1186/s13059-023-03059-9 (PMC10571256; doi:10.1186/s13059-023-03059-9)
Supplement: Supplementary file 2 — Additional file 2. Fig. S1. Expression of DRDD genes across tissues and relationship with methylation levels at Dynamic GbM genes. Fig. S2. Dynamic GbM methylation levels across 100 Arabidopsis accessions. Fig. S3. De novo methylation events in met1 mutants and WT segregants. Fig. S4. Example de novo methylation events in met1 mutant segregants at GbM loci. Fig. S5. RNA-seq analysis of Stable and Dynamic GbM genes. Fig. S6. Enrichment of histone acetylation in Dynamic and Stable GbM genes of different lengths. Fig. S7. Gene expression plasticity measured from high-throughput RNA-seq study of 54 tissue types. Fig. S8. Gene expression plasticity of genes closely linked to intergenic DRDD targets. Fig. S9. Enriched GO-terms in Dynamic and Stable GbM genes. Table S1. A list of all external datasets analyzed in this study. Table S2. Properties of EM-seq libraries sequenced in this study. [file 13059_2023_3059_MOESM2_ESM.pdf]

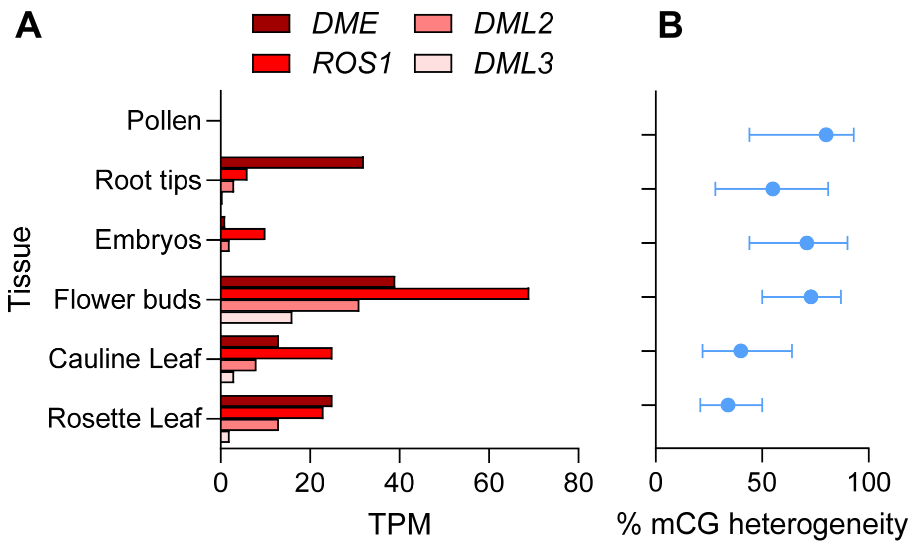

Figure S1: Expression of DRDD genes does not correlate with Dynamic GbM methylation heterogeneity. A) Average expression level of *DME, ROS1, DML2* and *DML3* in six tissue types measured by RNA-seq. B) CG methylation heterogeneity of Dynamic GbM genes in six tissue types. Dots represent median values, error bars represent interquartile range.

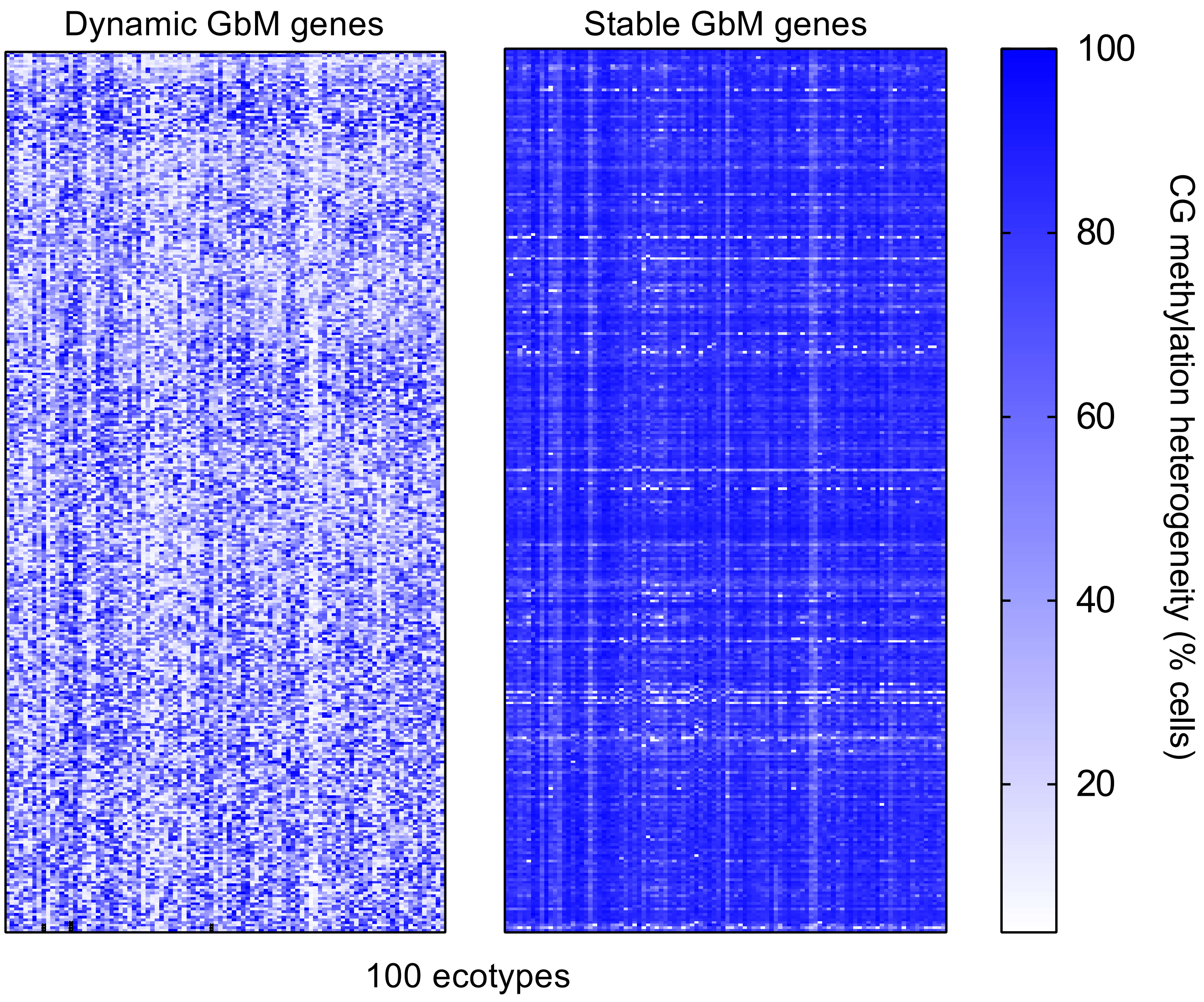

Figure S2: Dynamic GbM is consistent across Arabidopsis ecotypes. A heatmap showing CG methylation heterogeneity averaged across the gene bodies of Dynamic GbM and Stable GbM genes in 100 randomly selected ecotypes from the 1001 genomes project [34].

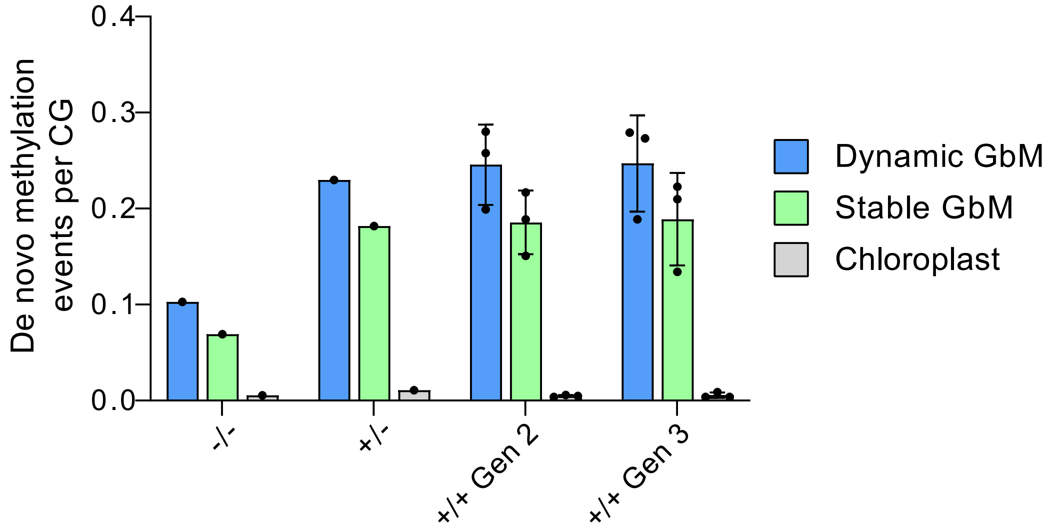

Figure S3: *De novo* methylation events in *met1* mutants and WT segregants. The rate of de novo CG methylation events per CG across all cells. Bars represent the mean, error bars represent standard deviation. Individual data points represent independent genetic lines that were sequenced. Chloroplast CG methylation is included as a control for the false positive rate from incomplete conversion or sequencing errors.

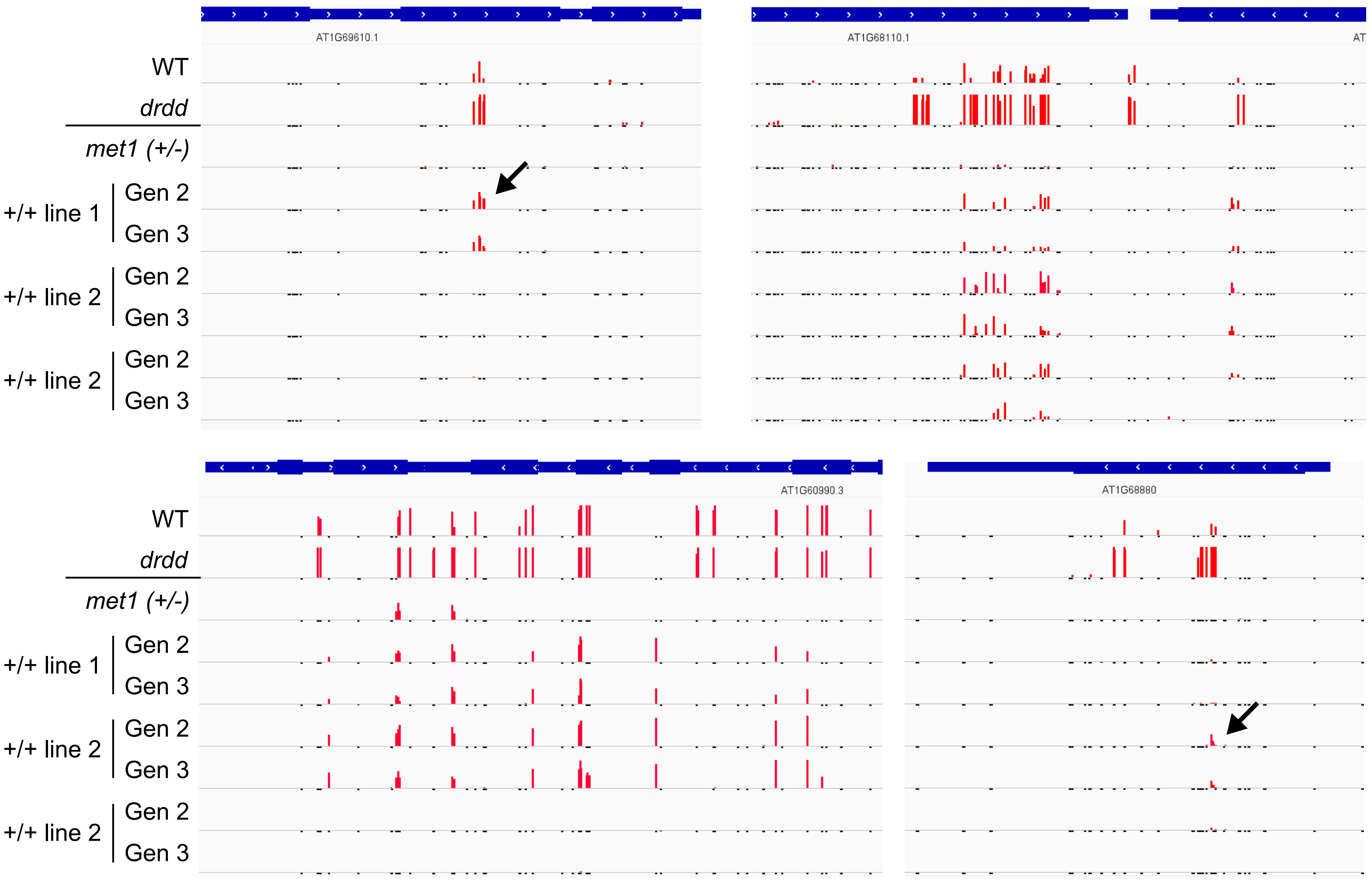

Figure S4: Example de novo methylation events in *met1* mutant segregants at GbM loci. Genome browser snapshots of four Dynamic GbM genes in WT, *drdd*, *met1* heterozygotes (+/-) and three independent lines of WT-segregant progeny (+/+).

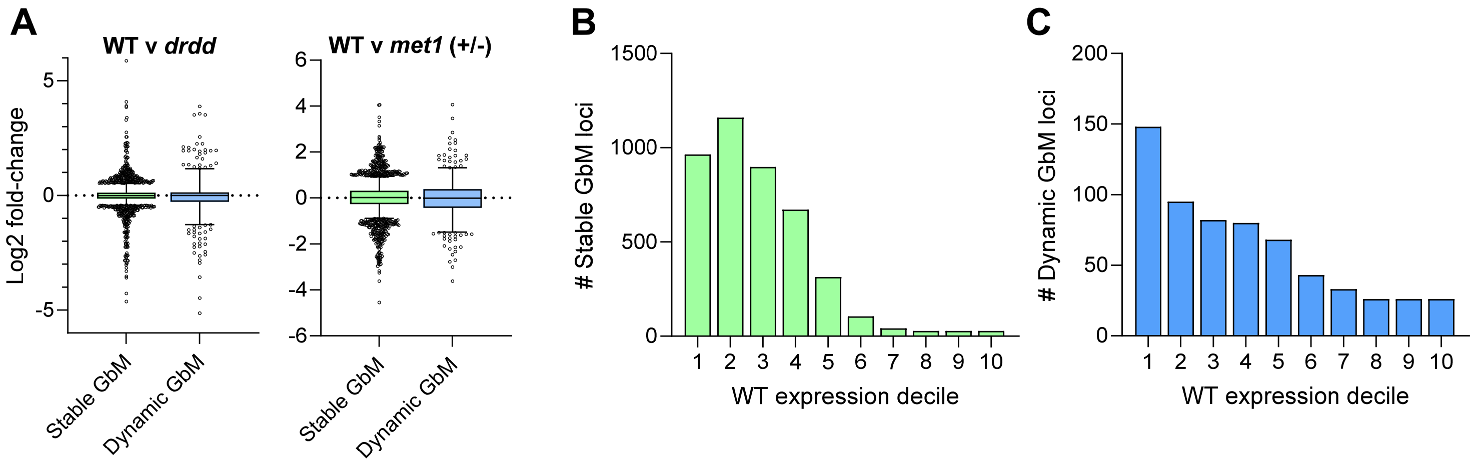

Figure S5: RNA-seq analysis of Stable and Dynamic GbM genes. A) Boxplots showing distribution of Log2 fold-change in transcript abundance for Stable and Dynamic GbM genes in WT v *met1* heterozygous mutants (which lose GbM) and WT v *drdd* mutants (which exhibit elevated GbM at Dynamic GbM genes). Horizontal line = median, box = interquartile range, whiskers = 1st and 99th percentile. B) Distribution of Stable GbM genes across WT transcript abundance deciles. C) Distribution of Dynamic GbM genes across WT transcript abundance deciles. In B and C, 1 = highest expression decile, 10 = lowest expression decile.

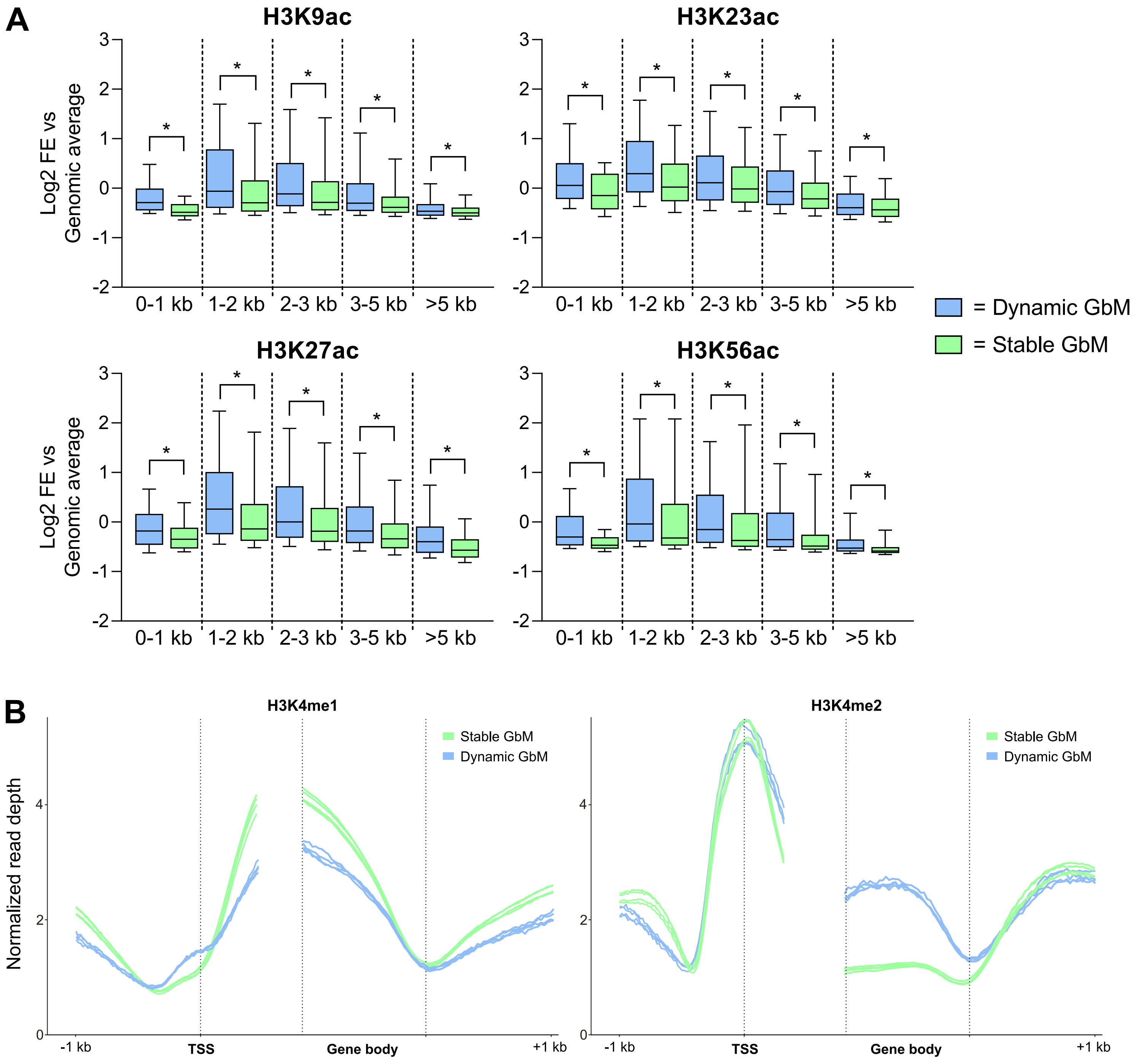

Figure S6: Enrichment of histone acetylation in Dynamic and Stable GbM genes of different lengths. A) Boxplots show the log2 fold read depth enrichment of four histone acetylation marks relative to the genomic average (ChIP-seq data were obtained from the plant chromatin state database[69]). Enrichment values are averaged across the whole coding region for each gene. Horizontal line = median, boxes = interquartile range, whiskers = 5th & 95th percentile. *p = <0.0001 (two-tailed parametric t-test). B) Smoothed moving average plots of ChIP-seq normalized read depth for H3K4me1 and H3K4me2 at Dynamic and Stable GbM genes. Individual lines represent ChIP-seq profiles from independent studies of WT Arabidopsis, obtained from the plant chromatin state database.

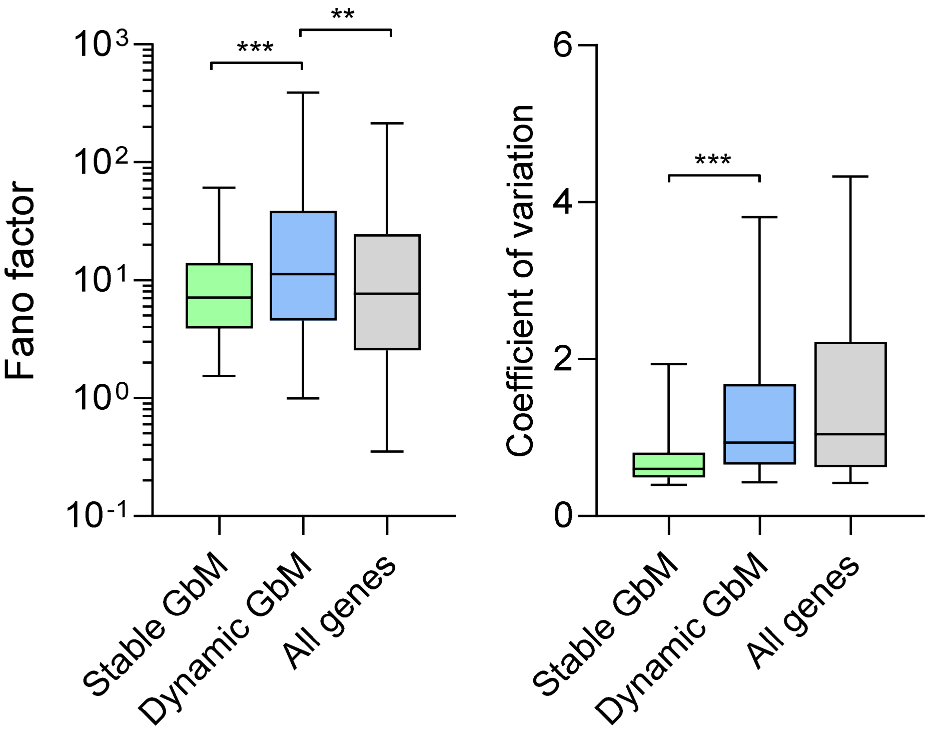

Figure S7: Gene expression plasticity measured from high-throughput RNA-seq study of 54 tissue types. Boxplots denote gene expression variability of Dynamic GbM, Stable GbM and total genes measured by the Fano factor (left) and Coefficient of variation (right). Transcript per million values of genes across 54 tissues were obtained from Mergner et al, 2020 [49]. Horizontal lines = median, boxes = interquartile range, whiskers = 5th & 95th percentile. ***p = < 0.0005, **p = < 0.005 (two-tailed parametric t-test).

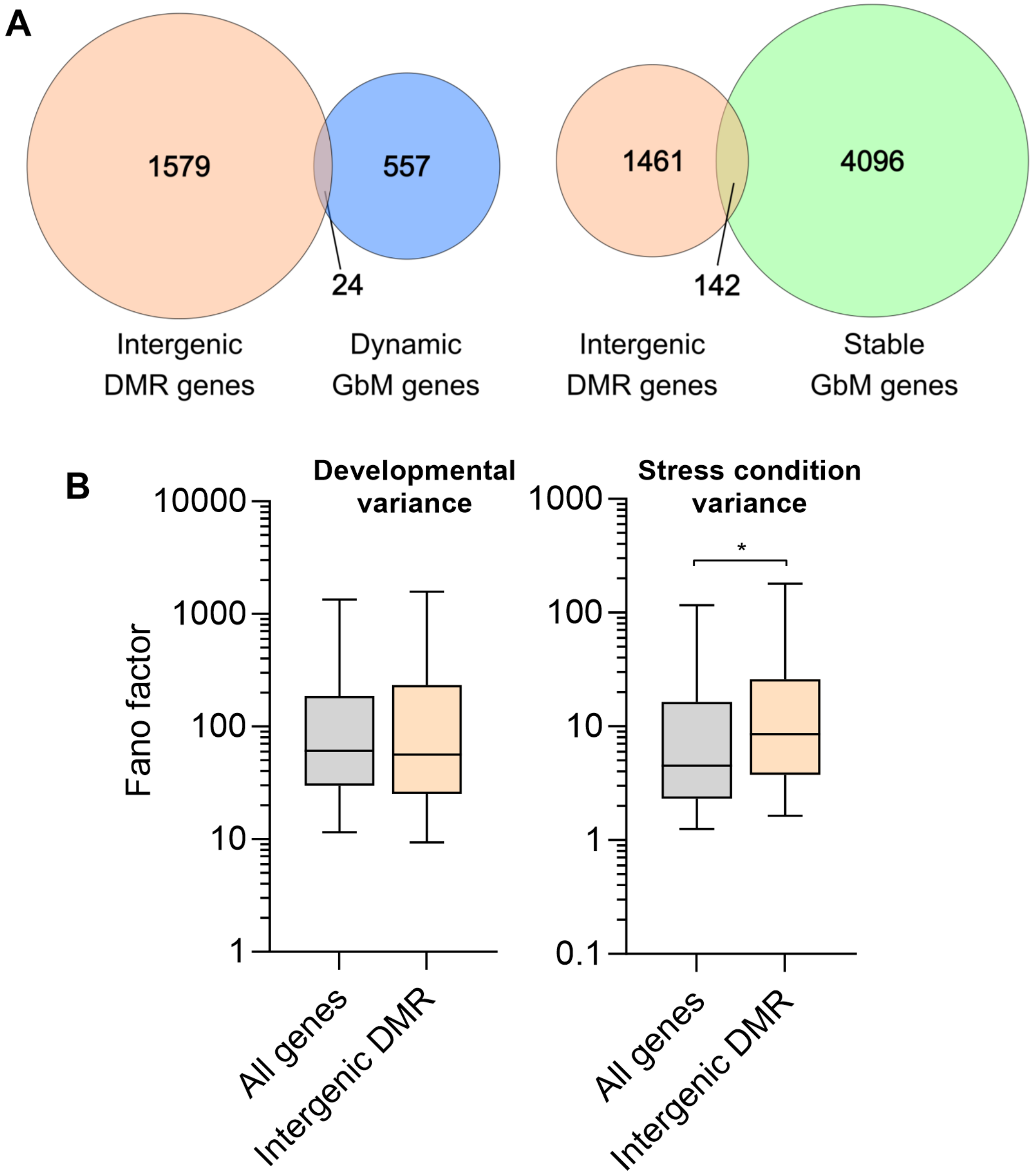

Figure S8: Gene expression plasticity of genes closely linked to intergenic DRDD targets.
A) Venn diagrams showing overlap between intergenic DMR genes (defined as a gene harboring a DRDD-target differentially methylated region in intergenic sequences within 2kb upstream or downstream of the coding region) and Dynamic or Stable GbM genes. B) Boxplots displaying gene expression variability as measured by the Fano factor across diverse developmental stages (left) and diverse physiological/stress conditions (right). Horizontal lines = median, boxes = interquartile range, whiskers = 5th & 95th percentile. *p = < 0.05 (two-tailed parametric t-test).

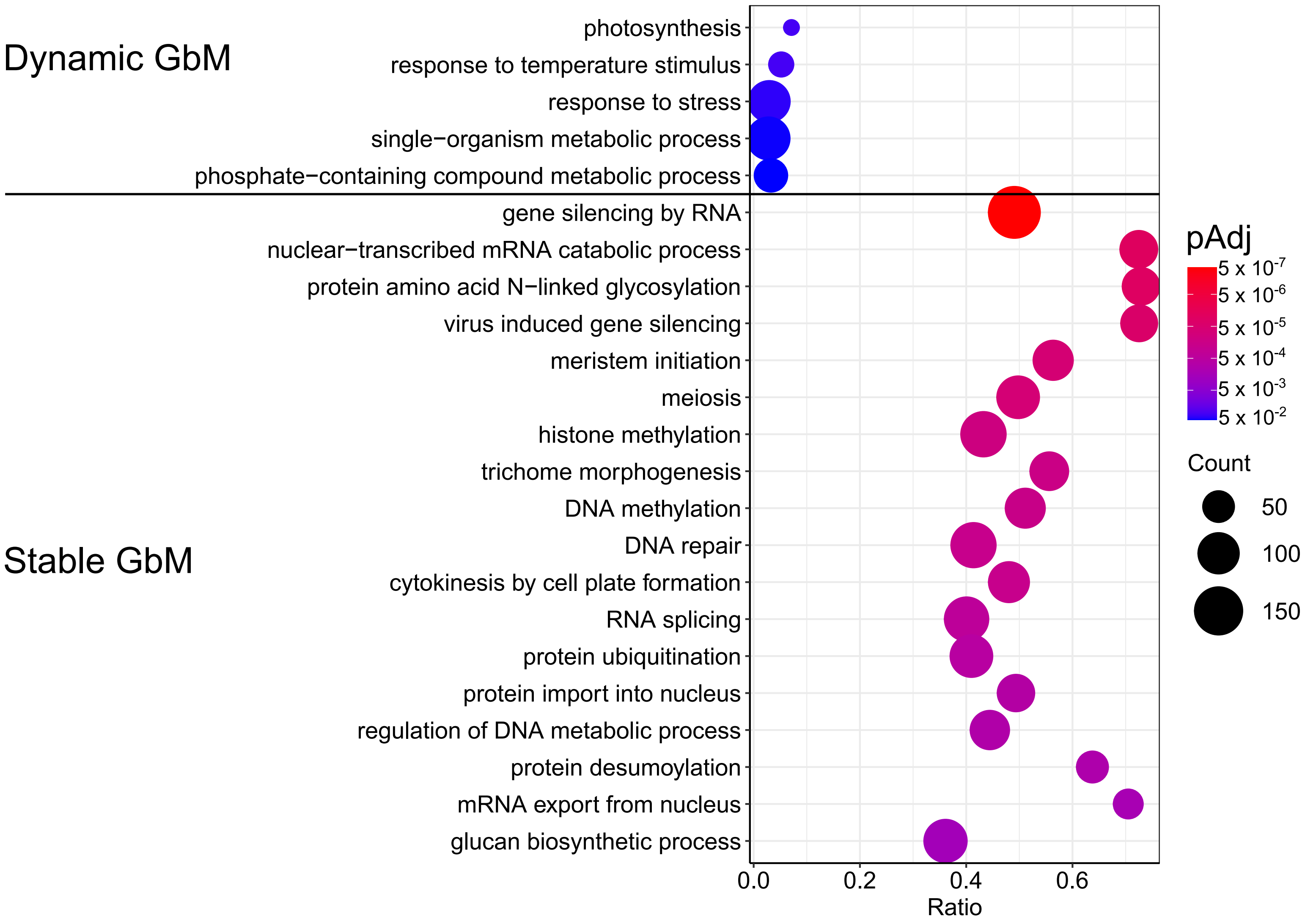

Figure S9: Enriched GO-terms in Dynamic and Stable GbM genes. Symbol color is a heat map scale for statistical significance and symbol size is proportional to the number of genes for the GO term. The x-axis indicates the enrichment ratio for each GbM category relative to all genes with the GO term.

Table S1: A list of all external datasets analyzed in this study

Original Study	Publication doi	Raw data accession
Bewick, A.J. et al, (2016) PNAS. 113 (32) 9111-9116	10.1073/pnas.1604666113	NCBI GEO GSE75071
Bourguet P. et al, (2021) Nat. Commun. 12:2683	10.1038/s41467-021-22993-5	NCBI GEO GSE146948
Chen, L.Q. et al, (2017) Plant J. 174(3):1795-1806	10.1104/pp.16.01944	NCBI GEO GSE73972
Gutzat, R. et al, (2020) EMBO J. 39:e103667	10.15252/embj.2019103667	EMBL EBI E-MTAB-5478 & E-MTAB-5479
Kawakatsu T., et al (2016). Nat Plants. 2:16058	10.1038/nplants.2016.58	NCBI GEO GSE79710
Kawakatsu, T. et al, (2016) Cell. 166(2):492-505	10.1016/j.cell.2016.06.044	NCBI GEO GSE43857
Khouider, S. et al, (2021) Nat. Commun. 12:420	10.1038/s41467-020-20606-1	NCBI GEO GSE141154
Liang, W. et al, (2022) New Phytol. 233:722–737	doi.org/10.1111/nph.17804	NCBI SRA PRJNA686693
Liu, Y. et al, (2018) Nucleic Acids. Res. 46:157-167	doi: 10.1093/nar/gkx919	http://systemsbiology.cau.edu.cn/chromstates
Long, J. et al, (2021) Science. 373:eabh0556	10.1126/science.abh0556	NCBI GEO GSE161625
Mergner J. et al, (2020) Nature, 579:409-14	10.1038/s41586-020-2094-2	https://www.nature.com/articles/s41597-020-00678-w
Niederhuth, C.E. et al, (2016) Genome Biol. 17:194	10.1186/s13059-016-1059-0	NCBI GEO GSE79526
Rigal, M. et al, (2016) PNAS. 113 (14) E2083-E2092	10.1073/pnas.1600672113	EMBL EBI PRJEB9919
Seymour, D.K. et al, (2014) PloS Genet. 10: e1004785	10.1371/journal.pgen.1004785	EMBL EBI PRJEB6701
Shang J-Y. et al, (2022) J. Integr. Plant Biol. 64:2438-54	10.1111/jipb.13406	NCBI GEO GSE206688
Stroud, H. et al, (2013) Cell. 152(1-2):352-64	10.1016/j.cell.2012.10.054	NCBI GEO GSE39901 & GSE38286
Stroud, H. et al, (2014) Nat. Struct. Mol. Biol. 21:64-72	10.1038/nsmb.2735	NCBI GEO GSE51304
Williams B.P., et al (2022). Plant Cell;34:1189–206	10.1093/plcell/koab319	NCBI GEO GSE191307

Table S2: properties of EM-seq libraries sequenced in this study

Sample	Tissue	Cycles	Total reads	Total reads after QC	Uniquely mapping reads after deduplication	% Reads uniquely mapping	Conversion rate
met1-3 heterozygote	leaf	2 x 150 bp	60,407,565	57,704,567	30,318,827	52.6	99.998
met1-3 WT segregant, line 1 F2	leaf	2 x 150 bp	59,741,235	56,772,575	32,355,357	57.1	99.9978
met1-3 WT segregant, line 1 F3	leaf	2 x 150 bp	53,407,468	53,362,670	22,649,572	42.4	99.999
met1-3 WT segregant, line 2 F2	leaf	2 x 150 bp	50,522,623	50,465,956	25,117,695	40	99.9989
met1-3 WT segregant, line 2 F3	leaf	2 x 150 bp	57,417,384	55,601,549	32,596,017	58.6	99.9978
met1-3 WT segregant, line 3 F2	leaf	2 x 150 bp	54,398,483	54,337,350	24,958,077	45.9	99.999
met1-3 WT segregant, line 3 F3	leaf	2 x 150 bp	55,237,611	55,193,429	26,779,936	48.5	99.9989
