# Supplementary material for: Dynamic DNA methylation turnover in gene bodies is associated with enhanced gene expression plasticity in plants
Source: Genome Biol. 2023 Oct 12;24:227. doi: 10.1186/s13059-023-03059-9 (PMC10571256; doi:10.1186/s13059-023-03059-9)
Supplement: Supplementary file 4 — Additional file 4. Review history. [file 13059_2023_3059_MOESM4_ESM.docx]

Review History

**First round of review**

**Reviewer 1**

**Were you able to assess all statistics in the manuscript, including the appropriateness of statistical tests used?**

Yes, and I have assessed the statistics in my report.

**Comments to author:**

The manuscript entitled "Dynamic DNA methylation turnover in gene bodies is associated with enhanced gene expression plasticity" describes a detailed analysis of the 'dynamic GbM', which is an extension of their previous paper "Somatic DNA demethylation generates tissue-specific methylation states and impacts flowering time" published in the Plant Cell, 2022.

The authors discovered dynamic GbM, increased CG methylation in drdd mutants. Dynamic GbM demethylated by DRDD demethylation pathway and methylated most likely by MET1. The authors described several exciting aspects of dynamic GbM: varying DNA methylation levels of dynamic GbM in somatic and germ line tissues, their evolutional conservation, associated histone markers, and their effect on transcription, including gene expression plasticity. Although this is an intriguing view on the role of GbM, the conclusion could be more persuasive with a few more analyses.

Major comments:

1. According to the authors' previous publication (Williams et al., Plant Cell, 2022, 34(4)1189-1206), 11% of genes with hypermethylated GbM in drdd plants were suppressed. However, the authors could not find an association between DNA methylation levels and gene expression levels in this study (up or down-regulation). Therefore, it is necessary to investigate why there is a discrepancy between the previous publication and the results described here.

2. The authors suggest that the dynamic GbM genes are more responsive to the stress or developmental cue (Figure 6), potentially due to their lower GbM levels than stable GbM genes (Figure 6B). If so, the authors could analyze the expression variance (wt vs. drdd) under various developmental stages or conditions (ex. Biotic, abiotic stress condition) rather than compare the steady-state variance among dynamic GbM genes. This analysis could further fortify the authors' model that the dynamic GbM affects gene expression plasticity (or regulatory potential).

3. The authors suggest that dynamic GbM is associated with gene expression plasticity. Previous papers reported that DNA demethylation at promoter transposable elements (Le et al., Genome Biol, 2014, 15:458), promoter-regulatory regions (Halter et al., eLife, 2021, 10:e62994) regulates responsive gene expression. The authors also showed that dynamic GbM largely overlapped with the promoter chromatin state (Figure 5). These previous reports and the promoter chromatin state of dynamic GbM suggest that DNA demethylation in the promoter, including DNA demethylation in transposable elements, is important for gene expression plasticity. There are possibilities that DNA methylation changes in the promoter, transposable elements are crucial for gene expression plasticity, and dynamic GbM in drdd is correlated with dynamic DNA methylation changes in the promoter, transposable elements in drdd. To support the authors' model, investigations of DNA methylation changes and gene expression plasticity concerning transposable elements and promoters in drdd are required.

Minor comments:

1. In figure 2, meristem and germline cells have higher DNA methylation levels at dynamic GbM genes than somatic cells Figure 2B and C). The germline cells have low H1 levels, which suppress DNA methylation. Also, the DNA methylation levels at dynamic GbM in somatic tissue (Figure 3B) appear lower than in germline cells (Figure 2C). Thus, H1 and DNA demethylases may collaborate to suppress dynamic GbM. Therefore, using public data, it will be much more informative if they could compare histone H1 levels at stable GbM genes vs. dynamic GbM genes to test whether H1 enriches at dynamic GbM.

2. In Figure 2, CG methylation heterogeneity levels of dynamic GbM vary among tissues. For example, the authors could check that CG methylation heterogeneity levels are anti-correlated with expression levels of DRDD among tissues.

3. Genes with GbM and H2A.Z shows higher responsiveness than conventional GbM genes (Coleman-Derr D et al., Plos Genet, 2012, 8(10): e1002988). Other than histone H3 modifications, it is necessary to investigate the level of H2 variants (e.g., H2A.Z) at dynamic GbM genes.

4. In Figure 5A, the authors should perform statistical analysis among dynamic GbM, stable GbM, and total exons in each histone mark.

5. The authors showed DNA methylation levels of some dynamic GbM genes are consistent in four ecotypes (Figure S1), but should not be limited to these ecotypes by further analyzing how many dynamic GbM genes consistently exist among natural variants from public data (Kawakatsu et al., Cell, 2016,166(2):492-505; Schmitz et al., Nature, 2013,495(7440):193-198)

6. The authors showed GO in dynamic GbM genes (Figure S5). It would be comprehensive if they could also analyze GO in stable GbM genes and compare it with GO in dynamic GbM genes. Also, the authors described as "enrichment ratio for DEGs" in the figure legend, which is incorrect as dynamic GbM genes were analyzed, not DEG (probably differentially expressed genes).

7. In page4, line 26,

"we compared the 581 genes most evidently targeted by DRDD to" in manuscript, but 582 genes in Figure 1B on Dynamic

8. In page5, line 8

"As the methylation state of these methylated genes are stable across development,"

There is no reference or supporting results for the above description.

9. In page10, line 15

"Figure S3" seems to be a typo of Figure S4

10. The authors need to describe the statistical test method in the figure legend and Method part.

11. The authors need to provide accession ID for previously published sequencing data in this manuscript.

**Reviewer 2**

**Were you able to assess all statistics in the manuscript, including the appropriateness of statistical tests used?**

Yes, and I have assessed the statistics in my report.

**Comments to author:**

The manuscript entitled: "Dynamic DNA methylation turnover in gene bodies is associated with enhanced gene expression plasticity" by Williams et al., reports the identification of genomic regions (protein coding genes: PCG) exhibiting particular profile of gene body methylation (GbM).

The authors determined, using 2 cutoffs, the DNA methylation pattern of PCG that gain CG methylation in the drdd plants. Such GbM pattern has been defined as dynamic GbM.

The authors address an interesting question and shed the light on genomic regions exhibiting particular epigenomic features.

The paper is well written and provide all the necessary information

The authors attempted to decipher the mechanisms controlling such type of GbM. Based on their conclusions it looks that complex processes regulate this type of DNA methylation pattern. Interestingly, the authors found that several histone marks overlap with these regions.

It seems that K36me3 content differs significantly between dynamic and stable GbM exons.

Any relationship with POL II and elongation ? (see questions below)

I would suggest to go a little be more into details in the analysis to fully cover all the

Few suggestions:

Are there small RNA (21-, 22- and 24-nt) in the identified genomic regions?

What is the DNA methylation pattern in rdr6, nrpb2, dcl234 plants ?

What is the DNA methylation pattern in sdg2, sdg8 plants ?

Are transcripts isoforms under the influence of the GbM?

Is DNA methylation more preferentially located in exons or in introns ?

Minor comments

Figure 5E : in all graphs the dot blot of stable loci is shown before the dynamic loci. Please invert.

I suggest also to provide a xls file with the AGI numbers of the stable and dynamic loci

**Reviewer 3**

**Were you able to assess all statistics in the manuscript, including the appropriateness of statistical tests used?**

Yes, and I have assessed the statistics in my report.

**Comments to author:**

The manuscript of "Dynamic DNA methylation turnover in gene bodies is associated with enhanced gene expression plasticity" by Williams et al, described the identification of subset of genes whit dynamic gene-body methylation (GbM) in arabidopsis, its evolution across distant lineages and potential association to gene expression. For a long time, the potential functions of GbM are not well understood. Williams' data provided new insights into this issue. However, the data here, especially the quality of figures was poor. Some key information/details were not included. It is a good start point to address the association between dynamic GbM and gene expression. But the manuscript in current status does not merit publication in GB.

I am pointing out my concerns need to be addressed.

1. poor quality and details in figures.

Fig.1

Fig.1A: lack of scales of CG/CHG/CHH methylation. Although I thought it should be 0-1 or 0-100%, but authors need to clearly label it.

Fig.1B: from the Venn, # of gene with Dynamic GbM is 582, but in the context the number was 581? And more, I think these 581 genes deserve a supplemental table with detailed information.

Fig.1C: the color of the boxplot showing stable and dynamic GbM is not consistent with Fig.1A, B and also other figures.

Fig.1D: maybe I did not understand it very well. I did not find out how to do the violin plot of methylation heterogeneity in methods. Authors might need to provide more details either in Fig. legends or in methods. And here why 10-85% was set as the cutoff?

Fig.2

Fig.2A: the genome browsers were not using the same scale (e.g. SAM). And actually, the genome browsers (IGV) images may be better saved in the svg format and then it can be easily formatted in illustrator. The same issues were also seen in Fig.3A, Fig.5F.

Fig.2A: the case-by-case presentation was acceptable, however, not just here, authors should also provide statistical presentation of the data at global/genome-wide level. For an example, how the global CG DNA methylation looks like in WT and drdd mutant in leaves.

Fig.4

Fig.4A: here better to add gene model of AT1G65810 in Arabidopsis and its homolog in other species.

Fig.5

In addition to H3K4me1/2, H3K36me3, H3K27ac and maybe H3K27ac also showed similar patterns as H3K4me1/2, authors need to further check their associations to dynamic GbM.

Fig.6

Fig.6B: the working model (right part) does not match the results/conclusion that dynamic GbM might be established via an unknown DNA methylation pathway not through MET1.

2. the association between dynamic GbM with H3K4me1/2 is weak.

Although we see difference between stable and dynamic GbM, it still hard to conclude that dynamic GbM is associated with H3K4me1/2.

In addition, if the association existed, CG methylation level/status should be further checked in H3K4me1/2/3 mutant and WT to validate authors' hypothesis.

3 the potential functions of genes with dynamic GbM were not clear.

the link of dynamic GbM to gene expression plasticity was poor.

4. lack the details of key information/tables/methods (not all of them here).

as mentioned in Fig.1B, the 581 genes with dynamic GbM should be grouped in supplemental table with details.

no summary of high through-put sequencing data, such as EM-seq, RNA-seq, DEGs.

no list/summary of altered transcripts in drdd and other mutants (vs WT).

not find the homologs of 581 genes with dynamic GbM in other 5 additional species.

data deposited in GEO not available.

EM-seq part, (paired-end 150x150 bp), here what 150x150 means? The same issue in Supplemental table 1.

As mentioned in Fig.1D, details in methods in violin plot for methylation heterogeneity should be provided.

if possible, version information of software/tools used in this study should be provided.

**Authors Response**

**Point-by-point responses to the reviewers’ comments:**

Reviewer #1:

The manuscript entitled "Dynamic DNA methylation turnover in gene bodies is associated with enhanced gene expression plasticity" describes a detailed analysis of the 'dynamic GbM', which is an extension of their previous paper "Somatic DNA demethylation generates tissue-specific methylation states and impacts flowering time" published in the Plant Cell, 2022.

The authors discovered dynamic GbM, increased CG methylation in drdd mutants. Dynamic GbM demethylated by DRDD demethylation pathway and methylated most likely by MET1. The authors described several exciting aspects of dynamic GbM: varying DNA methylation levels of dynamic GbM in somatic and germ line tissues, their evolutional conservation, associated histone markers, and their effect on transcription, including gene expression plasticity. Although this is an intriguing view on the role of GbM, the conclusion could be more persuasive with a few more analyses.

Major comments:

1. According to the authors' previous publication (Williams et al., Plant Cell, 2022, 34(4)1189-1206), 11% of genes with hypermethylated GbM in drdd plants were suppressed. However, the authors could not find an association between DNA methylation levels and gene expression levels in this study (up or down-regulation). Therefore, it is necessary to investigate why there is a discrepancy between the previous publication and the results described here.

*Reply: Thank you for pointing this out. We agree that the way this was written was confusing and contradictory. When comparing Dynamic GbM genes between WT and drdd, a fraction of those genes (~8%) are differentially expressed. This enrichment is significant compared to that expected by chance (hypergeometric test). As this comment points out, 11% of downregulated genes in drdd overlap CG gene body DMRs, which is also greater than expected by chance.*

*In our first submission of this manuscript, we concluded that collectively, Dynamic GbM gene expression is unaltered in drdd, as the majority of Dynamic GbM genes (~90%) show small (outside typical RNA-seq significance thresholds), so a large change to expression level is the exception rather than the rule when Dynamic GbM is abolished. However, we have now worded this section of the manuscript more accurately to reflect that there are significant changes to gene expression of a subset of these genes, and the boxplots in Figure S4 now also shows outlier points, to make the presence of differentially expressed genes more clear to readers. As you will see in our following comment, a number of the expression changes in drdd (including some that are non-significant) can be explained by a reduction in gene expression dynamic range.*

2. The authors suggest that the dynamic GbM genes are more responsive to the stress or developmental cue (Figure 6), potentially due to their lower GbM levels than stable GbM genes (Figure 6B). If so, the authors could analyze the expression variance (wt vs. drdd) under various developmental stages or conditions (ex. Biotic, abiotic stress condition) rather than compare the steady-state variance among dynamic GbM genes. This analysis could further fortify the authors' model that the dynamic GbM affects gene expression plasticity (or regulatory potential).

*Reply: We have significantly strengthened this part of the paper. We wish to note that our calculations of plasticity are performed with two large datasets. First, plasticity was calculated using microarray datasets with >160 samples for both developmental variability and environmental variability. Second, in this revised manuscript, we verified our findings by repeating the analysis with a more recent RNA-seq profile of 54 different tissue types (Mergner et al, 2020).*

*In order to accurately estimate gene expression plasticity in drdd mutants, we would need to repeat a similarly broad study of transcriptomes throughout development, which we feel is a large enough effort* *to be a study in its own right. However, while analyzing the gene expression plasticity of genes across development, we came up with a simple predictive model that could be tested with our existing drdd data. Many Dynamic GbM genes are at the extremes of the distribution of expression states in WT leaves vs other tissues. We reasoned that if DRDD directly enables a greater dynamic range of expression states, then drdd mutant leaves should show expression values closer to the mean expression state of all tissues compared to WT (as measured by Mergner et al, 2020). For example, if a Dynamic GbM gene in WT is very highly expressed in mature leaves (relative to other tissues), then drdd mature leaves should show reduced expression, whereas WT genes suppressed in mature leaves will be elevated in drdd.*

*To test this prediction we plotted the fold-change enrichment in WT leaves vs other tissues, and compared that to the fold change in drdd leaves vs WT. Consistent with our hypothesis there is a clear and highly significant negative slope. E.g. expression levels in drdd are much more likely to exhibit an expression change consistent with reduced gene expression plasticity. We have presented this new analysis in Figure 6, and we believe it offers strong support of the main conclusion of the paper, and is consistent with a causal link between demethylation by DRDD and enhanced gene expression plasticity. As a control, we show that this relationship does not exist for Stable GbM genes.*

3. The authors suggest that dynamic GbM is associated with gene expression plasticity. Previous papers reported that DNA demethylation at promoter transposable elements (Le et al., Genome Biol, 2014, 15:458), promoter-regulatory regions (Halter et al., eLife, 2021, 10:e62994) regulates responsive gene expression. The authors also showed that dynamic GbM largely overlapped with the promoter chromatin state (Figure 5). These previous reports and the promoter chromatin state of dynamic GbM suggest that DNA demethylation in the promoter, including DNA demethylation in transposable elements, is important for gene expression plasticity. There are possibilities that DNA methylation changes in the promoter, transposable elements are crucial for gene expression plasticity, and dynamic GbM in drdd is correlated with dynamic DNA methylation changes in the promoter, transposable elements in drdd. To support the authors' model, investigations of DNA methylation changes and gene expression plasticity concerning transposable elements and promoters in drdd are required.

*Reply: We provide two lines of evidence that the regulation of gene expression by promoter methylation and GbM patterns are separate phenomena. First, we show that there is almost no overlap between Dynamic GbM genes and genes that have a DRDD-target DMR within 2kb upstream or downstream (the same is true for Stable GbM genes) of their coding region. While Dynamic GbM genes have promoter-like chromatin, we see no evidence for their regulation by DNA methylation in the promoter. Secondly, we examine the gene expression plasticity of the genes associated with DRDD-target DMRs in their intergenic regions. In contrast to Dynamic GbM genes, intergenic DMR genes exhibit almost identical gene expression plasticity over development to the genomic average. Interestingly, intergenic DMR genes do show increased plasticity over diverse stress conditions, which is consistent with the literature (including those studies pointed out above) showing promoter methylation impacts the expression of biotic and abiotic stress genes, many of which are proximal to TEs. It’s therefore possible that DNA methylation levels in adjacent intergenic regions can impact plasticity, but we do not see strong evidence that Dynamic GbM is an extension of this mechanism.*

*We have presented our new analysis on intergenic-DMR associated genes in a new Supplemental Figure (Figure S8).*

Minor comments:

1. In figure 2, meristem and germline cells have higher DNA methylation levels at dynamic GbM genes than somatic cells Figure 2B and C). The germline cells have low H1 levels, which suppress DNA methylation. Also, the DNA methylation levels at dynamic GbM in somatic tissue (Figure 3B) appear lower than in germline cells (Figure 2C). Thus, H1 and DNA demethylases may collaborate to suppress dynamic GbM. Therefore, using public data, it will be much more informative if they could compare histone H1 levels at stable GbM genes vs. dynamic GbM genes to test whether H1 enriches at dynamic GbM.

*Reply: We find no H1 enrichment at either Dynamic or Stable GbM genes (data from two H1 ChIP-seq studies has been added to Figure 5A). We are not certain why germline and meristem cells possess higher methylation levels at Dynamic GbM, but we think it’s likely that developmental differences (e.g. cell cycle stage) contribute to this, as well as potentially more robust methylation pathway activity in these tissues.*

2. In Figure 2, CG methylation heterogeneity levels of dynamic GbM vary among tissues. For example, the authors could check that CG methylation heterogeneity levels are anti-correlated with expression levels of DRDD among tissues.

*Reply: We do not see a clear relationship between DRDD transcript levels and CG methylation heterogeneity. The variation in CG methylation between tissues is likely caused by a combination of factors including rates of cell division, activity of methyltransferases and DRDD expression.*

*The DRDD genes are actually most highly expressed in flower buds (a more highly methylated tissue), which also exhibit increased non-CG methylation and thus likely have the strongest “pushback” from methylation pathways. DRDD expression is low in pollen which might explain the slightly increased methylation heterogeneity in meiocytes and sperm cells.*

*Our analysis to address this question is presented as a Supplementary Figure (Figure S1).*

3. Genes with GbM and H2A.Z shows higher responsiveness than conventional GbM genes (Coleman-Derr D et al., Plos Genet, 2012, 8(10): e1002988). Other than histone H3 modifications, it is necessary to investigate the level of H2 variants (e.g., H2A.Z) at dynamic GbM genes.

*Reply: Figure 5A now includes H2A.Z for Dynamic GbM, Stable GbM and Total exons. The association between H2A.Z and Dynamic GbM is somewhat unclear. A minority of Dynamic GbM genes show strong enrichment for H2A.Z (likely overlapping with the genes considered “highly responsive” by Colman-Derr & Zilberman), but the majority show no H2A.Z enrichment relative to the genomic average.*

*By contrast, Stable GbM genes are depleted for H2A.Z, leading to a strong difference (D = >0.8) between Dynamic and Stable GbM genes, but a negligible difference between Dynamic GbM and total exons (D = ~0.1).*

*We did not find any association between other histone variants H2A.W and H2A.X and GbM genes. We have opted not to include these histone variants in Figure 5A, as the available ChIP-seq data is of lower quality than the other marks presented (only one replicate, and poor read depth in genic regions).*

4. In Figure 5A, the authors should perform statistical analysis among dynamic GbM, stable GbM, and total exons in each histone mark.

*Reply: These boxplots contain values for single exons and thus have very large sample sizes. As a consequence, almost all histone marks have a very low p-value, even after multiple hypothesis correction. As a more stringent measure of difference, we decided to annotate effect size measured by Cohen’s D, as we find the low p-values associated with these differences to be misleading (e.g. almost every histone mark is “significant” even if the effect size is small). We believe this statistical test is more stringent and more relevant to the data in question.*

5. The authors showed DNA methylation levels of some dynamic GbM genes are consistent in four ecotypes (Figure S1), but should not be limited to these ecotypes by further analyzing how many dynamic GbM genes consistently exist among natural variants from public data (Kawakatsu et al., Cell, 2016,166(2):492-505; Schmitz et al., Nature, 2013,495(7440):193-198).

*Reply: We have analyzed the methylation heterogeneity of Dynamic and Stable genes for 100 randomly selected ecotypes, using data downloaded from Kawakatsu et al (2016). These data are presented in a new heatmap in Figure S1, and they confirm that Dynamic GbM genes are heterogeneously methylated in all ecotypes, whereas Stable GbM genes exhibit high levels of methylation in all ecotypes.*

6. The authors showed GO in dynamic GbM genes (Figure S5). It would be comprehensive if they could also analyze GO in stable GbM genes and compare it with GO in dynamic GbM genes. Also, the authors described as "enrichment ratio for DEGs" in the figure legend, which is incorrect as dynamic GbM genes were analyzed, not DEG (probably differentially expressed genes).

*Reply: We have updated this supplemental figure to show the GO terms for Stable GbM genes as well.*

*We believe this GO analysis is consistent with the main model proposed in this paper. Stable GbM genes are enriched for many cellular housekeeping GO functions, whereas Dynamic GbM genes are enriched for functions more likely to be associated with high gene expression plasticity.*

7. In page4, line 26, "we compared the 581 genes most evidently targeted by DRDD to" in manuscript, but 582 genes in Figure 1B on Dynamic

*Reply: Thank you for pointing out this discrepancy, we have updated Figure 1 to show 581 Dynamic GbM genes.*

8. In page5, line 8 "As the methylation state of these methylated genes are stable across development," There is no reference or supporting results for the above description.

*Reply: We have amended this confusing sentence to be more clear. It now reads: “As the methylation state of these genes is consistent and stable across cells”.*

9. In page10, line 15 "Figure S3" seems to be a typo of Figure S4

*Reply: Thank you, this has been corrected.*

10. The authors need to describe the statistical test method in the figure legend and Method part.

*Reply: We have added more information on statistical methods in the figure legend and methods.*

11. The authors need to provide accession ID for previously published sequencing data in this manuscript.

*Reply: We have provided a new supplemental table (Table S1) with the citations, doi numbers and sequence read archive accession numbers for all external datasets used in this study.*

Reviewer #2: The manuscript entitled: "Dynamic DNA methylation turnover in gene bodies is associated with enhanced gene expression plasticity" by Williams et al., reports the identification of genomic regions (protein coding genes: PCG) exhibiting particular profile of gene body methylation (GbM).

The authors determined, using 2 cutoffs, the DNA methylation pattern of PCG that gain CG methylation in the drdd plants. Such GbM pattern has been defined as dynamic GbM. The authors address an interesting question and shed the light on genomic regions exhibiting particular epigenomic features.

The paper is well written and provide all the necessary information. The authors attempted to decipher the mechanisms controlling such type of GbM. Based on their conclusions it looks that complex processes regulate this type of DNA methylation pattern. Interestingly, the authors found that several histone marks overlap with these regions.

It seems that K36me3 content differs significantly between dynamic and stable GbM exons.

Any relationship with POL II and elongation ? (see questions below)

*Reply: We agree that understanding the relationship between Pol II elongation and GbM states is an interesting question. One hypothesis we tested is whether either GbM state is associated with high or low rates of Pol II pausing, using the raw data from this paper:*

[*https://www.nature.com/articles/s41477-018-0280-0*](https://www.nature.com/articles/s41477-018-0280-0)*. We found that neither Stable nor Dynamic GbM genes have a statistically significant association with either the paused or non-paused genes. In fact, the amount of overlap between GbM and transcriptional pausing is so low that we can assert this is a negative result with some confidence.*

I would suggest to go a little be more into details in the analysis to fully cover all the Few suggestions:

Are there small RNA (21-, 22- and 24-nt) in the identified genomic regions?

*Reply: We checked two small RNA datasets for the presence of small RNAs in Dynamic GbM genes: Lunardon et al 2020* [*(https://genome.cshlp.org/content/30/3/497*](https://genome.cshlp.org/content/30/3/497)*) and Erdmann et al, 2017. In both datasets, we found almost 0 association between 21-24nt small RNAs and Dynamic GbM gene coding regions. We have opted not to present this data in the manuscript as we don’t believe it adds to the story, but we are open to feedback if the reviewers feel this will be useful data to include.*

What is the DNA methylation pattern in rdr6, nrpb2, dcl234 plants?

*Reply: We mapped the methylation data for these mutants (from Stroud et al, 2013) and analyzed their methylation patterns at Dynamic GbM genes. Both rdr6 and dcl234 show methylation patterns completely indistinguishable from WT, and these data have been added to the other mutants in Fig 3.*

*Nrpb2 mutants showed a significant but very small effect reduction in methylation compared to WT. We are cautious to conclude anything from this as the effect size is small (~3-5% lower genic methylation compared to WT!). This effect is tiny compared to the bigger methylation changes in nrpb2 reported by Stroud et al, 2013, so we are not convinced methylation at Dynamic GbM loci is strongly dependent on the “slow Pol II” mutation of nrpb2 that has been profiled.*

What is the DNA methylation pattern in sdg2, sdg8 plants?

*Reply: We also mapped the methylation profiles of these mutants. sdg8 mutants show no methylation differences at Dynamic GbM genes, but interestingly sdg2 mutants showed substantially increased methylation levels. As SDG2 is an H3K4 methyltransferase, we found this result interesting. We therefore also analyzed the methylation profiles of COMPASS complex atx1;2;4;5 mutants, which also exhibit reduced H3K4me2 and H3K4me3 (using data from*

*Shang et al, 2022:* [*https://onlinelibrary.wiley.com/doi/full/10.1111/jipb.13406*](https://onlinelibrary.wiley.com/doi/full/10.1111/jipb.13406)*). In these mutants, we also found elevated DNA methylation levels at Dynamic GbM genes. These data are consistent with a regulatory interaction between DRDD and H3K4me2/3, and we have added this to Figure 5. Shang et al also pointed out that the expression of ROS1, DML2 and DML3 are somewhat reduced in atx1;2;4;5 mutants, but we do not believe this is the reason for increased methylation at Dynamic GbM genes, as we do not see similar increases at other DRDD targets (i.e. intergenic regions). These data have been included as a new panel in Figure 5.*

Are transcripts isoforms under the influence of the GbM?

*Reply: We have looked extensively at splicing variation and whether or not GbM states overlap isoform diversity and we have not found a relationship between either.*

Is DNA methylation more preferentially located in exons or in introns?

*Reply: Both Dynamic and Stable GbM are marginally enriched in exons compared to introns. Since this enrichment in exons has already been described for gene body methylation generally (Takuno & Gout, 2011, Mol. Biol. Evol.), we have opted not to include it, but we are happy to do so if the reviewers think this is important.*

Minor comments

Figure 5E: in all graphs the dot blot of stable loci is shown before the dynamic loci. Please invert.

*Reply: This has been corrected.*

I suggest also to provide a xls file with the AGI numbers of the stable and dynamic loci.

*Reply: We have provided a supplementary table with the gene IDs of the Stable and Dynamic GbM loci (Table S3).*

Reviewer #3:

The manuscript of "Dynamic DNA methylation turnover in gene bodies is associated with enhanced gene expression plasticity" by Williams et al, described the identification of subset of genes whit dynamic gene-body methylation (GbM) in arabidopsis, its evolution across distant lineages and potential association to gene expression. For a long time, the potential functions of GbM are not well understood. Williams' data provided new insights into this issue. However, the data here, especially the quality of figures was poor. Some key information/details were not included. It is a good start point to address the association between dynamic GbM and gene expression. But the manuscript in current status does not merit publication in GB.

I am pointing out my concerns need to be addressed.

1. poor quality and details in figures.

Fig.1

\# Fig.1A: lack of scales of CG/CHG/CHH methylation. Although I thought it should be 0-1 or 0-100%, but authors need to clearly label it.

*Reply: Thank you for bringing this to our attention, this has been fixed. All browser snapshot scales are 0-100% and we have specified this in the legend.*

Fig.1B: from the Venn, # of gene with Dynamic GbM is 582, but in the context the number was 581? And more, I think these 581 genes deserve a supplemental table with detailed information.

*Reply: Thank you for pointing out the discrepancy. We have fixed the venn diagram to show 581 genes. We have also provided a supplementary table listing the gene IDs of these 581 genes.*

Fig.1C: the color of the boxplot showing stable and dynamic GbM is not consistent with Fig.1A, B and also other figures.

*Reply: Thank you for bringing this to our attention, we have corrected the colors to be consistent with other figures.*

Fig.1D: maybe I did not understand it very well. I did not find out how to do the violin plot of methylation heterogeneity in methods. Authors might need to provide more details either in Fig. legends or in methods. And here why 10-85% was set as the cutoff?

*Reply: We have added details on how methylation heterogeneity was calculated and how the violin plot was generated to the methods. The cutoffs of 10-85% were chosen as the profile of the violin plot showed the majority of genomic CGs exhibit methylation heterogeneity values outside of this range. The majority of genomic CGs are almost completely unmethylated (<10%) or fully methylated (>85%). CGs targeted by DRDD exhibiting intermediate methylation are therefore unusual, and our cutoffs were designed to isolate these from the typical genomic pattern.*

Fig.2

Fig.2A: the genome browsers were not using the same scale (e.g. SAM). And actually, the genome browsers (IGV) images may be better saved in the svg format and then it can be easily formatted in illustrator. The same issues were also seen in Fig.3A, Fig.5F.

*Reply: We are so grateful for this suggestion! We were previously unaware that IGV could export vector images, and we agree that our previous figure resolution was poor. We have updated all figures to contain svg browser snapshots.*

Fig.2A: the case-by-case presentation was acceptable, however, not just here, authors should also provide statistical presentation of the data at global/genome-wide level. For an example, how the global CG DNA methylation looks like in WT and drdd mutant in leaves.

*Reply: We would like to point at that a thorough analysis of global/genome-wide methylation differences between WT and drdd is presented in our previous paper (Williams et al, 2022, Plant Cell). In this study we identified all differentially methylated regions in every sequence context.*

*We have also added more details at the beginning of the results section of the manuscript to alert readers to our previous results, and clarify that the global difference in CG levels between WT and drdd is very small (23.9% (WT) vs 25.5% (drdd)).*

Fig.4

Fig.4A: here better to add gene model of AT1G65810 in Arabidopsis and its homolog in other species.

*Reply: Thank you for this suggestion, we have added gene models for all homologs to this figure.*

Fig.5

In addition to H3K4me1/2, H3K36me3, H3K27ac and maybe H3K27ac also showed similar patterns as H3K4me1/2, authors need to further check their associations to dynamic GbM.

*Reply: We have expanded our discussion of histone acetylation marks in the results and discussion sections. We have also included a new Supplemental Figure showing that the enrichment of four histone acetylation marks (H3K9ac, H3K23ac, H3K27ac and H3K56ac) within the gene bodies of Dynamic GbM genes is independent of gene length, much like H3K4me1.*

*H3K36me3 is enriched within Stable GbM genes which is typical of long and robustly transcribed genes. They are marginally depleted within Dynamic GbM genes, but this depletion is not substantial compared to total genes in the genome (Fig. 5A), and we observed no difference in DNA methylation between WT and sdg8 mutants, which harbor a loss of function mutation in the histone methyltransferase that deposits H3K36me3. We therefore do not feel we have sufficiently strong evidence to explore an association between Dynamic GbM and H3K36me3 levels further in this manuscript.*

Fig.6

Fig.6B: the working model (right part) does not match the results/conclusion that dynamic GbM might be established via an unknown DNA methylation pathway not through MET1.

*Reply: Thank you for pointing this out. We had intended to add a “?” to MET1 in the Dynamic GbM part of this model, but we had unintentionally left this out. This has now been corrected. We do believe that MET1 is the most likely candidate for de novo methylation of Dynamic GbM genes (hence why we suggest it’s activity in the working model) but we wish to leave room for doubt as the current data are not totally conclusive.*

2. the association between dynamic GbM with H3K4me1/2 is weak.

Although we see difference between stable and dynamic GbM, it still hard to conclude that dynamic GbM is associated with H3K4me1/2.

*Reply: We do not agree that the association between H3K4me1 and 2 is weak. We wish to point out that the antibody for H3K4me2 produces moderate levels of enrichment in all datasets, so the fold change enrichment may not appear strong compared to some other histone marks, but the levels of H3K4me2 at Dynamic GbM genes are comparable to high peaks of H3K4me2 that are observed in promoters. The mean Dynamic GbM coding region has higher H3K4me2 (averaged across the whole gene body) than 43% of promoters at their highest peak. This strongly suggests that Dynamic GbM coding regions exhibit promoter-like enrichment of this histone mark.*

*We also wish to point out that Figs. 5A and E plot the fold enrichment across all exons for Dynamic and Stable GbM genes. This brings the average level of Dynamic GbM enrichment down as not all exons of Dynamic GbM genes are evenly methylated and enriched for regulatory chromatin (see Fig. 5F as an example).*

In addition, if the association existed, CG methylation level/status should be further checked in H3K4me1/2/3 mutant and WT to validate authors' hypothesis.

*Reply: We agree that this is a good suggestion. We therefore analyzed the methylation levels of Dynamic GbM genes in 2 mutant backgrounds important for the methylation of H3K4: sdg2 and quadruple mutants atx1;2;4;5 (using data from Shang et al, 2022:* [*https://onlinelibrary.wiley.com/doi/full/10.1111/jipb.13406*](https://onlinelibrary.wiley.com/doi/full/10.1111/jipb.13406)*).*

*In both of these mutants, we observe substantially elevated DNA methylation levels at Dynamic*

*GbM genes. These data are consistent with a regulatory interaction between DRDD and H3K4me2/3. Shang et al also pointed out that the expression of ROS1, DML2 and DML3 are somewhat reduced in* *atx1;2;4;5 mutants, but we do not believe this is the reason for increased methylation at Dynamic GbM genes, as we do not see similar increases at other DRDD targets (i.e. intergenic regions). These data have been included as a new panel in Figure 5. These data are consistent with a regulatory association between H3K4me2 and Dynamic GbM.*

3 the potential functions of genes with dynamic GbM were not clear.

*Reply: We have included a more detailed discussion of the gene ontology functions associated with Dynamic and Stable GbM genes in the final results section of the manuscript, and updated the Supplementary Figure to also include Stable GbM GO term enrichment.*

the link of dynamic GbM to gene expression plasticity was poor.

*Reply: We have significantly strengthened this part of the paper. We wish to note that our calculations of plasticity are performed with two large datasets. First, plasticity was calculated using microarray datasets with >160 samples for both developmental variability and environmental variability. Second, in this revised manuscript, we verified our findings by repeating the analysis with a more recent RNA-seq profile of approximately 60 different tissue types (Mergner et al, Nature, 2020).*

*In order to accurately estimate gene expression plasticity in drdd mutants, we would need to repeat a similarly broad study of transcriptomes throughout development, which we feel is a large enough effort to be a study in its own right. However, while analyzing the gene expression plasticity of genes across development, we came up with a simple predictive model that could be tested with our existing drdd data. We reasoned that if DRDD directly enables a greater dynamic range of expression states, then drdd mutants should show expression values closer to the mean expression state of all tissues compared to WT (as measured by Mergner et al, 2020). For example, if a Dynamic GbM gene in WT is very highly expressed in mature leaves (relative to other tissues), then drdd mature leaves should show reduced expression, whereas WT genes suppressed in mature leaves will be elevated in drdd.*

*To test this prediction we plotted the fold-change enrichment in WT leaves vs other tissues, and compared that to the fold change in drdd leaves vs WT. Consistent with our hypothesis there is a clear and highly significant negative slope. E.g. expression levels in drdd are much more likely to exhibit an expression change consistent with reduced gene expression plasticity. We have presented this new analysis in Figure 6, and we believe it offers strong support of the main conclusion of the paper.*

4. lack the details of key information/tables/methods (not all of them here).

as mentioned in Fig.1B, the 581 genes with dynamic GbM should be grouped in supplemental table with details.

*Reply: We have provided a supplementary table with the gene IDs of Dynamic and Stable GbM genes.*

no summary of high through-put sequencing data, such as EM-seq, RNA-seq, DEGs.

*Reply: A summary of EM-seq data is presented in Supplementary Table I. All RNA-seq information, including a list of DEGs is presented in a previous paper: Williams et al, 2022, Plant Cell.*

no list/summary of altered transcripts in *drdd* and other mutants (vs WT).

*Reply: A comprehensive analysis of altered RNA seq patterns between drdd and WT is presented in a previous paper: Williams et al, 2022, Plant Cell.*

not find the homologs of 581 genes with dynamic GbM in other 5 additional species.

*Reply: We have included a new Supplemental Table with the homology search results for all five species, which includes the Arabidopsis gene ID, the gene ID of the closest homolog, the homology relationship (e.g. one-to-one, one-to-many etc) the Phytozome ortholog group number, and whether or not the homolog was included in the conservation analysis used in Figure 4 (only one-to-one orthologs were considered reliable for this section of the paper.*

data deposited in GEO not available.

*Reply: We will deposit all data for this study on GEO upon acceptance of the publication.*

EM-seq part, (paired-end 150x150 bp), here what 150x150 means? The same issue in Supplemental table 1.

*Reply: We have corrected this table (now supplemental table 2) to more clearly denote paired end 150 x 150 bp.*

As mentioned in Fig.1D, details in methods in violin plot for methylation heterogeneity should be provided.

*Reply: We have added this information to the methods.*

if possible, version information of software/tools used in this study should be provided.

*Reply: We have added software version information to the methods as well.*

**Second round of review**

**Reviewer 1**

During the revision process, the authors strengthened the manuscript and provided additional data to support the potential functions of GbM in gene expression. The revised main text and figures are clear to read and understand. Overall, these changes have greatly improved the manuscript.

Although the authors mostly responded to the comments from the previous review, the following comments should be addressed before publication.

1. The authors suggest that the dynamic GbM genes are more responsive to the stress or developmental cue, potentially due to their lower GbM levels than stable GbM genes cue (Figure 6). To support this, the authors have added new analysis data of gene expression fold change between WT leaves vs. other tissues and compared this to the fold change in drdd leaves vs. WT in revised Fig 6E. Although there is a significant negative correlation, the R2 value is 0.145, which is marginal. To suggest that dynamic GbM genes are more responsive to the stress or developmental cue, the authors could check the gene expression variance of several dynamic GbM genes related to stress response or developmental process between drdd vs. WT under stress or developmental stages.

2. To prove that the regulation of gene expression by promoter methylation and GbM patterns are separate phenomena, the authors analyzed DRDD-target DMRs in intergenic regions (revised Fig. S8). In response, the authors mentioned that "we show that there is almost no overlap between Dynamic GbM genes and genes that have a DRDD-target DMR within 2kb upstream or downstream (the same is true for Stable GbM genes) of their coding region". Taken together with revised Fig. S8, the authors may mean to define the intergenic region as 2kb upstream and/or downstream of the coding region. However, the definition of the intergenic region is missing from the revised manuscript and the figure legend. The authors need to clarify this.

3. The authors mentioned gene body methylation with different chromatin contexts in the discussion. Although the results suggest that H2A.Z-rich gene body methylation may be different from the active gene body methylation genes identified here, the authors could analyze the overlap between H2A.Z-rich gene body methylated genes (Coleman-Derr et al., PLOS Genetics 8(10): e1002988, 2012), ddm1-dependent gene body methylated genes (Lee et al., Genome Biology, 24:106, 2023), epimutation hot spots (Hazarika et al., Nature Plants. 8(2):146-156, 2022), and genes with dynamic gene body methylation. This comparison would provide valuable information on how different (or similar) these gene body methylation subtypes are.

**Reviewer 2**

The authors clarified all the points and add new analyses that strengthen their findings.

**Reviewer 3**

The revised version of "Dynamic DNA methylation turnover in gene bodies is associated with enhanced gene expression plasticity" by Williams et al, presented a much better improvement. Authors added genetic and genomic data linking the dynamic GbM and H3K4me1/2 and more data for explanation of how the dynamic GbM impacts on gene expression plasticity. Most of my concerns have been responded or provided detailed interpretation.

Some minor concerns:

For Fig.5B, which 4 types of chromatin states used for analysis are not clear, and here why authors use a different set of data, not the same set of data from Fig.5A?

A similar question for Fig.5C, which set of data are used for the analysis?

For Fig.5F we saw relative high H3K4me2 levels on the gene bodies of Dynamic GbM while that is in gene regulatory regions of Stable GbM. In addition to the case-by-case example, how the globally H3K4me2 profiles looks like across genes with Dynamic GbM (vs genes with Stable GbM)? I think this might make the data more strong.

In Table S2, for Cycles 150x150bp, is it 2x150bp presenting the paired-end 150bp sequencing?

**Authors Response**

**Point-by-point responses to the reviewers’ comments:**

Reviewer #1:

During the revision process, the authors strengthened the manuscript and provided additional data to support the potential functions of GbM in gene expression. The revised main text and figures are clear to read and understand. Overall, these changes have greatly improved the manuscript.

Although the authors mostly responded to the comments from the previous review, the following comments should be addressed before publication.

1. The authors suggest that the dynamic GbM genes are more responsive to the stress or developmental cue, potentially due to their lower GbM levels than stable GbM genes cue (Figure 6). To support this, the authors have added new analysis data of gene expression fold change between WT leaves vs. other tissues and compared this to the fold change in drdd leaves vs. WT in revised Fig 6E. Although there is a significant negative correlation, the R2 value is 0.145, which is marginal. To suggest that dynamic GbM genes are more responsive to the stress or developmental cue, the authors could check the gene expression variance of several dynamic GbM genes related to stress response or developmental process between drdd vs. WT under stress or developmental stages.

*Reply: We wish to point out that Fig. 6E presents a linear regression model rather than a correlation analysis. A Pearson correlation analysis points to a fairly strong relationship with r = - 0.4. While the linear regression model R2 value of 0.145 might seem low, we believe it is a reasonable and expected result given the context of the model. Our model predicts 14.5% of the expression changes between WT and drdd solely from the dynamic range of gene expression over WT development. Given that drdd is a complex and pleiotropic mutant, harboring a large number of expression changes relating to diverse biological functions (see our previous paper) we believe that 14.5% of the variance being explained by loss of gene expression plasticity is a notable result that is not marginal. We also believe the strong statistical significance and the absence of this signal in other GbM genes (Fig. 6F) suggests this result is robust. We have added some wording to the discussion to better explain the results of this simple model. We recognize that experimentally defining gene expression variance in mutants would be valuable, but to do this robustly will represent a major body of work that we believe is more appropriate for an additional follow up study.*

2. To prove that the regulation of gene expression by promoter methylation and GbM patterns are separate phenomena, the authors analyzed DRDD-target DMRs in intergenic regions (revised Fig. S8). In response, the authors mentioned that "we show that there is almost no overlap between Dynamic GbM genes and genes that have a DRDD-target DMR within 2kb upstream or downstream (the same is true for Stable GbM genes) of their coding region". Taken together with revised Fig. S8, the authors may mean to define the intergenic region as 2kb upstream and/or downstream of the coding region. However, the definition of the intergenic region is missing from the revised manuscript and the figure legend. The authors need to clarify this.

*Reply: thank you for bringing this to our attention, we have now clarified the definition of intergenic regions in both the legend and the methods section of the manuscript.*

3. The authors mentioned gene body methylation with different chromatin contexts in the discussion. Although the results suggest that H2A.Z-rich gene body methylation may be different from the active gene body methylation genes identified here, the authors could analyze the overlap between H2A.Z-rich gene body methylated genes (Coleman-Derr et al., PLOS Genetics 8(10): e1002988, 2012), ddm1-dependent gene body methylated genes (Lee et al., Genome Biology, 24:106, 2023), epimutation hot spots (Hazarika et al., Nature Plants. 8(2):146-156, 2022), and genes with dynamic gene body methylation. This comparison would provide valuable information on how different (or similar) these gene body methylation subtypes are.

*Reply: Of the datasets suggested to overlap with our gene sets, only Colman-Derr & Zilberman published the complete list of H2A.Z genes. We have intersected this gene list with Stable and Dynamic GbM genes, and we find a minimal, non-significant overlap between both:*

*We have included the precise number of Dynamic GbM genes overlapping H2A.Z enriched genes in the relevant part of the results section of the manuscript.*

*The recent study on DDM1-associated GbM genes does not include a complete list of these loci. However, we note that this study analyzed all contexts of methylation within gene bodies, including non-CG and TE-like methylation. This is very different to our definition of GbM as a solely CG methylation defined epigenetic state that is distinct from transcriptional silencing pathways like RdDM. While their study includes a number of CG-methylated GbM genes that change methylation state in ddm1, you can see from Figure 3B & C of that paper that this is a very small subset of genes compared to the major methylation changes in ddm1, which are at loci methylated in all sequence contexts. We also do not fully understand the classification of GbM genes by this paper, as they define the total set of GbM genes in WT as >10,000 genes which is more than double the number that previous studies (and our study) have defined.*

*Unfortunately, Hazarika et al also did not publish the genomic coordinates or list the overlapping genes of the epimutation hotspots outlined in their paper. However, from reading their paper there are several clues that these hotspots are likely located within Stable GbM (rather than Dynamic GbM genes). Genes with epimutation hotspots are typically longer, and enriched for H3K4me1, similar to Stable GbM genes.*

**Reviewer 2**

The authors clarified all the points and add new analyses that strengthen their findings.

**Reviewer 3**

The revised version of "Dynamic DNA methylation turnover in gene bodies is associated with enhanced gene expression plasticity" by Williams et al, presented a much better improvement. Authors added genetic and genomic data linking the dynamic GbM and H3K4me1/2 and more data for explanation of how the dynamic GbM impacts on gene expression plasticity. Most of my concerns have been responded or provided detailed interpretation.

Some minor concerns:

For Fig.5B, which 4 types of chromatin states used for analysis are not clear, and here why authors use a different set of data, not the same set of data from Fig.5A?

*Reply: Thank you for bringing this to our attention, we have added more information on how we performed this chromatin state analysis in the manuscript and the methods. There have been multiple published methods to identify chromatin states from histone modification ChIPseq data. Many of these methods use similar or overlapping public datasets. We have based our chromatin state categories on those described by Sequeira-Mendes et al (2014), as in our experience the chromatin states defined in this paper are biologically meaningful. If one looks at the histone mark enrichment profiles for each chromatin state published by Sequeira-Mendes et al, one can clearly see the histone marks associated with “promoter” and “intergenic” chromatin are enriched in Dynamic GbM compared to Stable GbM in Fig. 5A.*

A similar question for Fig.5C, which set of data are used for the analysis?

*Reply: We have clarified in greater detail how we performed this analysis. It uses the same “distal promoter” chromatin state from Sequeira-Mendes et al (State 2 in that paper) as shown in Fig. 5B. Fig. 5C shows a similar result to Fig. 5B but shows overlap as the number of bp rather than % of gene body. We feel this is a useful addition to the figure as readers will intuitively grasp number of base pairs, and it is not affected by length differences between the coding regions of Stable and Dynamic GbM genes.*

For Fig.5F we saw relative high H3K4me2 levels on the gene bodies of Dynamic GbM while that is in gene regulatory regions of Stable GbM. In addition to the case-by-case example, how the globally H3K4me2 profiles looks like across genes with Dynamic GbM (vs genes with Stable GbM)? I think this might make the data more strong.

*Reply: While other panels in Figure 5 show the global levels of H3K4me2 at Dynamic and Stable GbM as boxplots, we agree that averaged profiles across Dynamic and Stable GbM genes are a useful addition. We have presented a smoothed moving average of H3K4me1 and H3K4me2 ChIP-seq read depth enrichment across the TSS and gene bodies of Dynamic and Stable GbM genes within Figure S6.*

In Table S2, for Cycles 150x150bp, is it 2x150bp presenting the paired-end 150bp sequencing?

*Reply: Yes. 2x150bp is less confusing so we have changed the wording to this.*

**Third round of review**

**Reviewer 1**

The author correctly addressed the questions raised in the previous version.

**Reviewer 3**

The authors clarified all my concerns.

**Authors Response**

**Point-by-point responses to the reviewers’ comments:**

*Reply:* *The authors wish to express thanks to all reviewers for their attention and hard work.*
